# Post-COVID-19 Illness Trend in a Local Community in Bangladesh

**DOI:** 10.7759/cureus.45998

**Published:** 2023-09-26

**Authors:** Md Fahad Hossain, Syed Nurul Aziz, Mahfuza Akter, Manish Kharel, Nitesh Mandal, Indresh Yadav, Anjali Mandal, Roshan Rajbanshi

**Affiliations:** 1 Internal Medicine, Jahurul Islam Medical College, Dhaka, BGD; 2 Hospital Medicine, Upazila Health Complex, Ministry of Health, Dhaka, BGD; 3 Internal Medicine, Shaheed Suhrawardy Medical College, Dhaka, BGD; 4 Medicine, Sylhet MAG (Muhammad Ataul Goni) Osmani Medical College, Sylhet, BGD; 5 Internal Medicine, Getwell Hospital, Biratnagar, NPL; 6 Internal Medicine, Samar Hospital and Research Center Pvt. Ltd., Janakpur, NPL; 7 Internal Medicine, Community Based Medical College, Mymensingh, BGD; 8 Medicine and Surgery, Chitwan Medical College, Bharatpur, NPL; 9 Medicine and Surgery, Nobel Medical College, Biratnagar, NPL

**Keywords:** risk factors, old age, post-covid symptoms, covid-19, clinical symptoms

## Abstract

Background: An infection with coronavirus disease 2019 (COVID-19) might show a wide range of symptoms. Many individuals still experience symptoms after a prolonged period of initial COVID-19.

Objectives: The objective is to find out the prolonged consequences of COVID-19 with their associations.

Materials and method: Two hundred and eighty-six COVID-19 cases were the subject of this cross-sectional investigation, which was carried out in basic and secondary healthcare facilities in Bangladesh. COVID-19-positive participants with consent were interviewed in person about their sociodemographic traits, the nature of their COVID-19 infection, risk factors, present manifestations, etc. We carried out our statistical exploration by use of IBM SPSS Statistics for Windows, Version 22 (Released 2013; IBM Corp., Armonk, New York, United States). To evaluate differences, we utilized the chi-square (χ2) test as well as the unpaired t-test. Our significance threshold level was 0.05.

Result: In this study, 18.5% of participants reported having post-COVID-19 symptoms. The four main symptom categories were anorexia (26.4%), myalgia (34.8%), fatigue (41.5%), and palpitations (25.5%). The majority of post-COVID-19 syndrome patients (e.g., 40.0%) were over 50 years old. Severe disease (81.8%) was more likely to develop post-COVID-19 illness.

Conclusion: Fifty-three out of 286 participants (or 18.5%) reported having post-COVID-19 symptoms. The main symptom categories included fatigue, myalgia, anorexia, and palpitations. In order to determine the risk variables our data supports, additional investigation is required.

## Introduction

Coronavirus disease 2019 (COVID-19) was first discovered in Wuhan state of China and is caused by SARS-CoV-2. It belongs to corona a family with genetic SARS virus similarity. A person with COVID-19 symptoms is referred to as a symptomatic case [1]. According to WHO's 2020 World Health Statistics, COVID-19 not only puts lives and livelihoods in danger but also threatens recent advancements in health and the achievement of the UN's sustainable development targets.

The t-cell-mediated immunological response is a significant immune response for COVID-19. The SARS-CoV investigation demonstrated that lung epithelial cells infected IL-8 and IL-6 from the virus [2]. COVID-19 patient's lungs showed significant inflammatory cell infiltration, and it is likely that these cells are a combination of the innate and adaptive system of immunity. The most common cell of innate immunity is likely to be neutrophils, according to our predictions. However, neutrophils are a double-edged sword since they can also cause lung damage. T cells usually make the major portion of adaptive immunity, but severe patients of COVID-19 have a marked decline. CD8+ cells are the primary cytotoxic T cells. However, T cells with pathogenic cytotoxic properties generated from CD4+ positive T cells are found in the worst cases [3].

Previous reports of 101 COVID-19 deceased cases, non-specific clinical signs, and common symptoms like fever, coughing, and dyspnea are all available. With the progression of the illness, acute-phase reactants gradually increased [4]. Previous epidemiological studies looked at the risk factors for worse outcomes. The multivariable analysis identified certain risk factors such as increasing age, elevated sum of SOFA (sequential organ failure assessment), and a level of d-dimer exceeding 1 g/mL upon admission. Atherosclerotic heart disease, hypertension, and diabetes were considered risk variables during the univariable analysis [5].

Research in the fields of epidemiology and virology suggests that the primary ways COVID-19 spreads are through close respiratory droplet exposure to symptomatic individuals, proximity to infected individuals, or touch of contaminated goods. Virological and clinical research found that SARS-CoV-2 spread most quickly during the beginning phase (the initial three days following symptom onset) and from the upper part of the respiratory system (nose and throat) by evaluating biological samples from confirmed COVID-19 patients [6-8]. The incubation time for COVID-19 typically lasts 5 to 6 days. From acquiring infection to 14 days called the "pre-symptomatic" phase during which infected people can still spread the disease [9].

Li et al. (2020) discovered advancing age along with underlying conditions such as diabetes, and high blood pressure raises mortality from COVID-19 pneumonia [10]. The risk of death is also increased with bacterial infections. In severe patients, malnutrition was widespread. Organ damage like lung, heart, kidney, and liver dysfunction were also common manifestations [10].

The influence of COVID-19 on several organ systems and its wide variety of enduring symptoms, including myalgia, weariness, dyspnea, coughing, and loss of taste and/or smell, are already well-known [11]. To categorize symptoms of proven COVID-19 infection after infection, a comprehensive post-COVID-19 symptom model has recently been put out. The symptoms were categorized by length of time there. After recovering from COVID-19, individuals typically experience short-term post-COVID-19 symptoms lasting 5 to 12 weeks, followed by medium-term post-COVID-19 issues that persist for 12 to 24 weeks. Additionally, some people may continue to experience prolonged symptoms, extending beyond 24 weeks [12]. The relapsing-remitting symptom pattern that many people with and without COVID-19 experience is represented in this model [13].

Nearly 1 million persons, or 1.6% of the population, self-reported post-COVID-19 symptoms during a four-week period in the UK, according to the study [14]. The poll also revealed that 18.5% of individuals reported having problems with their regular tasks as a result of the disease, indicating a negative challenge brought on by prolonged COVID-19 in daily activities. This should be treated seriously because many persons who experienced symptoms long after contracting COVID-19 indicated that they were unable to resume their earlier caliber of performance and were constantly burdened by the symptoms.

Research is necessary to determine the character, frequency, and length of post-COVID-19 symptoms encountered by affected individuals, as well as any relevant elements that might influence health care, to enhance patient management. This is particularly true for developing nations [15], like Bangladesh, where many people reside in rural areas. There have only been two published articles about persistent COVID-19 infection in Bangladesh. One was a survey of 1002 people of whom 20% reported having symptoms that persisted following COVID-19, with diarrhea (12.7%) and exhaustion (11.5%) being the two most prevalent symptoms [16]. A small research study involving 355 samples discovered that among the survivors of COVID-19, 46% still had symptoms, and the most commonly reported symptom was fatigue [17]. We wanted to find out how often post-COVID-19 illness occurs and to explore whether there are any possible associations between the reported symptoms and unrelated factors.

## Materials and methods

An observational study involving 286 COVID-19-positive cases was carried out. A gene expression test called real-time PCR was used to diagnose COVID-19-positive cases. People aged 18 years or more who recovered after therapy, went to the hospital for follow-up care, or later reported persisting symptoms met the inclusion criteria. Individuals who were too ill to participate, those who rejected consent, and those we were unable to reach were excluded. Following that, a sample size calculation was performed using parameters such as 50% of expected frequency, 5% level of significance or 95% confidence level, and degree of accuracy or tolerable error, which is often set at 5-10%. A minimum of 96 samples and a maximum of 384 samples were used to generate the sample size. Finally, 286 examples were chosen based on the criteria for selection. All patients who contracted COVID-19 were interviewed by researchers. A standardized questionnaire was prepared and used to collect the data from all the samples. The questionnaire was validated by the appropriate Government authority of that particular area. The questionnaire contains five sections. The first section includes five questions on socio-demographic characteristics (age, gender, employment, marital status, education level). The second section includes 15 questions about the symptoms the patient experienced during illness (fever, cough, weight loss, shortness of breath, fatigue, malaise, myalgia, headache, diarrhea, heart rate, and severity). The third section includes six questions for any comorbidities (HTN, diabetes mellitus, COPD, asthma, obesity, and others that include pregnancy, CKD, CLD, etc.). The fourth section (10 questions) Includes questions about the prevalence of post-COVID-19 illness and symptoms of post-COVID-19 illness. The fifth section (seven questions) includes the investigation findings of the patients. Data were collected using a data collection sheet. The face-to-face interviewer provided participants with a brief explanation of the study's methodology, a request for their voluntary participation, and a guarantee of the confidentiality of their information. The interview was conducted by multiple authors. The patients were interested in participating in the study voluntarily and stated that they did not have any issue with the data being used for scientific purposes. The protocol of identifying the contacts of COVID-19 persons who had previously been infected, creating contact with them, and gathering data during the pandemic of COVID-19 was ethically approved and permitted by the relevant authorities of the Bangladesh Government. For data processing, we made use of the computer applications IBM SPSS Statistics for Windows, Version 22 (Released 2013; IBM Corp., Armonk, New York, United States) and Microsoft Excel. Numeric data (quantitative data) are shown in terms of the average value along with the standard deviation (mean and standard deviation), while non-numeric data (qualitative data) are represented as the ratio and occurrence frequency (frequency and proportion). To tabulate and present the comparison results graphically, tables, histograms, pie charts, bar diagrams, graphs, and charts were employed.

## Results

**Figure 1:**
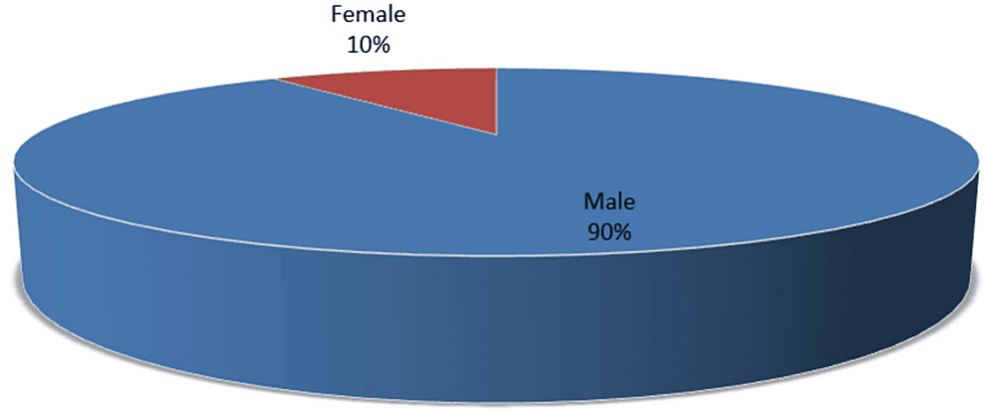
Gender breakdown of research participants (n=286)

The distribution of patients' ages is shown in Table 1. According to the survey, a major number of the patients (33.5%) fell in the 31-40 age range, followed by individuals in the 41-50 age range (26.2%). The patient was 39.2±5.7 years old on average. Two hundred and fifty-seven (89.86%) of the 286 cases had males and 29 (10.13%) had females. The ratio of men to women was 8.86:1 (Figure 1).

**Table 1:** Distribution by age (n=286)

Age (years)	Number of patients	Percentage (%)
≤30	45	15.7
31-40	96	33.5
41-50	75	26.2
51-60	48	16.7
61-70	22	7.6
Mean ± SD	39.2±5.7	

The distribution of cases by clinical manifestation is shown in Table 2. The most frequent diagnoses, occurring in 79.7% and 35.6% of patients, respectively, were fever and cough. Other symptoms were headache in 46 patients (16.0%), diarrhea in 57 patients (20.0%), tachycardia in 25 patients (8.7%), and quick breathing in 13 patients (4.5%). Thirty-eight patients in total were asymptomatic (Table 2).

**Table 2:** Case distribution based on clinical symptoms (n=286)

Clinical manifestation	Number of patients*	Percentage (%)
Fever	228	79.7
Cough	102	35.6
Dyspnea	59	20.6
Headache	46	16.0
Diarrhea	57	20.0
Asymptomatic	38	13.2
Tachycardia	25	8.7
RR > 30 breaths/min	13	4.5
SpO_2_		
> 90%	273	95.4
< 90%	13	4.5
Crepitation over lung	11	3.8
Hypotension	25	8.7

Diabetes (8%), hypertension (10%), asthma (4%), cardiovascular disease (2.4%), and five patients who were pregnant at different weeks (Table 3) were the co-morbidities.

**Table 3:** Patients' risk factor profiles (n=286)

Risk factors	Number of patients*	Percentage (%)
Hypertension	29	10.1
Diabetes mellitus	23	8.0
Asthma	11	3.8
IHD	7	2.4
Pregnancy	5	1.7

**Figure 2:**
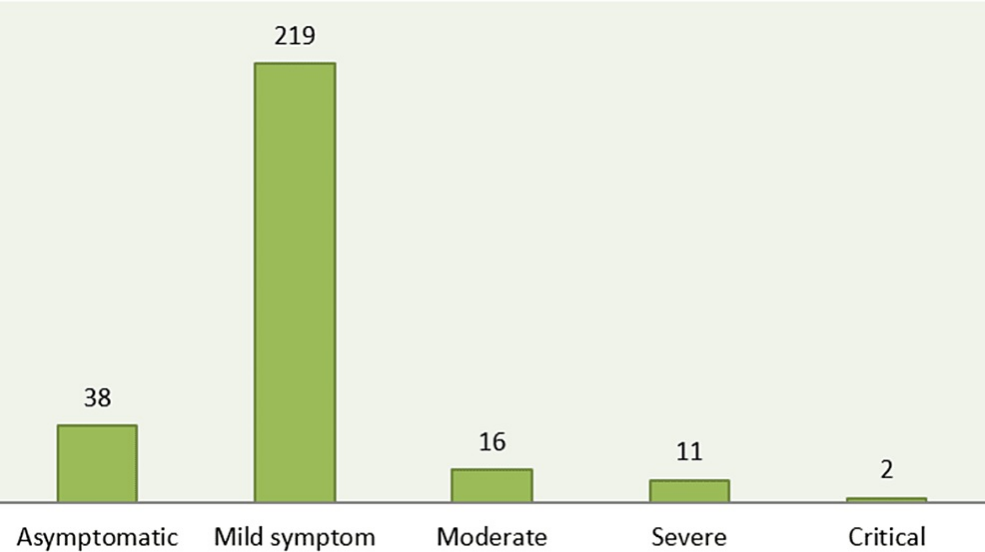
Clinical features of COVID-19 according to severity (n=286)

In this investigation, patients were split into five groups (Figure 2). Thirty-eight (13.2%) patients were asymptomatic, 219 (76.5%) had mild symptoms, 16 (5.59%) had moderate disease, 11 (3.8%) had severe symptoms, and 2 (0.69%) had critical symptoms.

In Table 4, the laboratory profile is shown. On a chest X-ray, it was discovered that there was one case of leucopenia, 25 cases of lymphopenia, 38 cases of lymphocytosis, 72 cases of CRP positivity, and 43 cases of pneumonitis.

**Table 4:** Patient's laboratory profiles (n=286)

Variables	Mean ±SD	Range
Hb % (gm/dl)	13.8 ± 1.6	10.5 – 15.3
TC of WBC (×10^9^)	6.8 ± 0.5	3.9 – 25.4
Neutrophil count (×10^9^)	58.3 ± 11.2	42.3 – 79.1
Lymphocyte count (×10^9^)	29.9 ± 7.5	20.4 – 38.8
Platelet count (×10^9^)	241.5 ± 74.2	186.7 – 291.2
CRP	23.2 ± 5.4	3.2 – 45.3
S. Creatinine	1.2 ± 0.4	0.7 – 3.2

**Figure 3:**
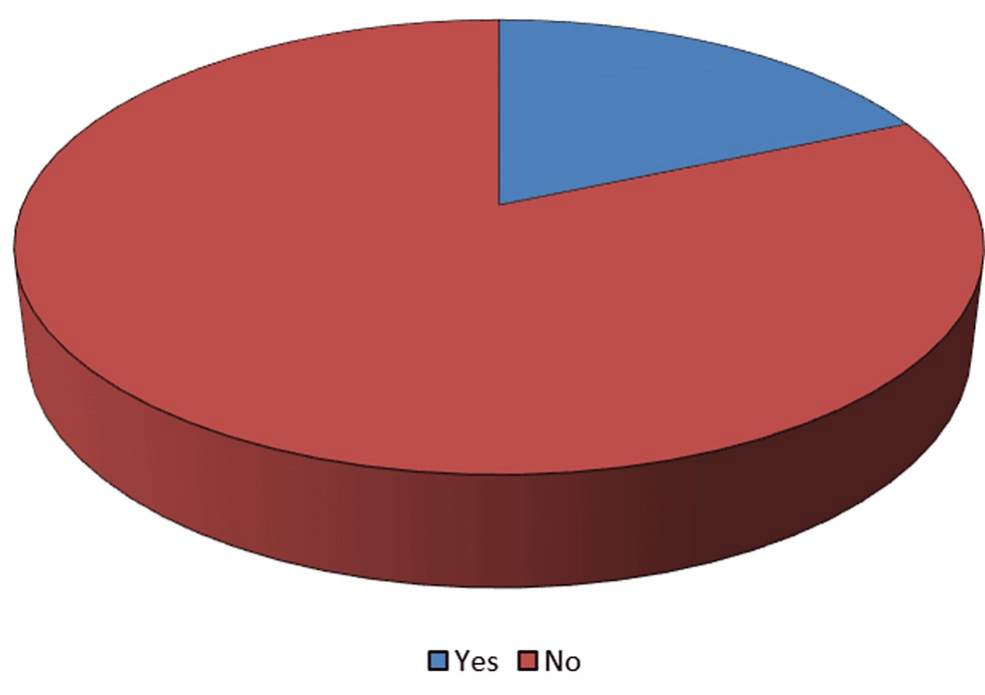
Frequency of post-COVID-19 symptoms (n=286)

The frequency of post-COVID-19 symptoms is depicted in Figure 3. Fifty-three patients, or 18.5%, in all, reported one or more symptoms. Therefore, 18.5% of people reported having post-COVID-19 symptoms.

The post-COVID-19 symptoms are displayed in Table 5. The four main symptom categories were anorexia (26.4%), myalgia (34.8%), fatigue (41.5%), and palpitations (25.5%).

**Table 5:** Distribution of incidents by their most prominent post-COVID-19 symptoms (n=53)

Post-COVID-19 symptoms	Number of patients*	Percentage (%)
Fatigue	22	41.5
Weakness	14	26.4
Myalgia	15	34.8
Anorexia	14	26.4
Palpitation	11	25.5
Cough	9	20.9
Insomnia	10	23.2

The many post-COVID-19 syndrome-related parameters are displayed in Table 6. Age, diabetes mellitus, and illness severity were identified as the risk factors in a univariate analysis. The age groups were separated into those under and over 50. Age >50 was a factor in the post-COVID-19 syndrome for the majority of patients (e.g., 40.0%); the difference was statistically significant, making the senior age a significant risk factor (p 0.05). Disease severity was observed, and the result showed that the patients with more severe disease (81.8%) had a considerable possibility of post-COVID-19 illness development.

**Table 6:** An analysis of the post-COVID-19 syndrome's risk variables in a single variable (n=286)

Variables	Total number of patients (n=286)	Post-COVID-19 symptoms (n=53)	Percentage (%)	p-value
Age				
<50 year	216	25	11.5	0.031
>50 year	70	28	40.0	
Sex				
Male	257	46	17.8	0.096
Female	29	7	24.1	
Diabetes Mellitus				
Yes	23	14	60.8	0.025
No	263	39	14.8	
Hypertension				
Yes	29	11	37.9	0.062
No	257	42	16.3	
Disease severity				
Mild	219	36	16.4	
Moderate	16	8	50.0	0.001
Severe	11	9	81.8	

## Discussion

The trends of COVID-19 with its symptoms and any potential associated risk factors are evaluated in this study. Among the 286 COVID-19-infected patients, 31-40 years (mean difference 39.2-5.7 years) were the most common age groups accounting for 33.5%. The trend was most common among the male. Gupta et al. supported our study in which the age group 21-35 years was frequent, especially among males, suggesting the presence of COVID-19 among male adults [18]. 

A systematic analysis by Rodriguez-Morales et al. in January and February 2020, based on 656 cases, revealed that increased temperature and cough were frequent presentations [19]. In a previous study as well, 94% of patients reported recent fever. In addition, cough (88%) and shortness of breath (81%) with diarrhea (6.3%) were other frequent findings [20]. Another study conducted in Bangladesh found that fever was the primary complaint in 77% of patients, followed by sore throat (12.5%), myalgia (12.5%), fatigue (7.5%), and diarrhea (3%) [21]. Similarly, in our study, raised temperature and cough are the frequent manifestations accounting for 79.7 % and 37.6% respectively. Headache, diarrhea, and shortness of breath are less frequent manifestations. It is worth noting that 13.2% were completely asymptomatic but asymptomatic individuals and carriers can still transmit the infection to others. This study also identifies diabetes (8%), hypertension (10%), asthma (4%), and cardiovascular disease (2.4%) as comorbidities, and five individuals were pregnant at various weeks which is consistent with earlier investigations [21]. In addition, very few severe and critical cases were identified in this study but a relation between comorbidities and the clinical outcomes is not demonstrated, which signifies the importance of further studies. 

This study also highlighted the prevalence and post-COVID-19 common manifestations and any risk factors for the occurrence of these symptoms. In this study, 18.5% of participants reported having post-COVID-19 symptoms which is consistent with Carson et al., in which long COVID-19 symptoms were found to be 16.1 % at 12 weeks [11]. Similarly, the study done in the UK also documented the 10 percent occurrence of long COVID-19 syndrome [22]. Among the 53 patients experiencing post-COVID-19 symptoms main symptom categories were anorexia (26.4%), myalgia (34.8%), fatigue (41.5%), and palpitations (25.5%) which is supported by another study. One study identified eight long-lasting COVID-19 symptoms, with fatigue ranking highest and muscle soreness and dyspnea coming in second and third, respectively [11]. In a different study [23], 14% of participants experienced fatigue issues between 3 and 9 months. Following this, several studies list shortness of breath as the second most frequent symptom [11,24], while some research lists anosmia, coughing, and myalgia as typical long-term COVID-19 symptoms [25]. Similarly, Davis et al. defined exhaustion, lethargy, and impaired cognition as the prevailing symptoms [26]. During the risk factor analysis, most of the post-COVID-19 patients (40.0%) were found over 50 years old which is statistically significant, making the advanced age a significant risk factor for the condition. In addition, the severity of the disease was also found to be one of the statistically significant elements for the persistence of symptoms accounting for 88% (p:0.05). Moreover, this study also highlighted diabetes as another significant risk factor whereas gender and hypertension are statistically insignificant in association with post-COVID-19 symptoms. Therefore, it can be concluded that the Likelihood of persistence of post-COVID-19 increases with age and disease severity. 

This study highlighted the trend of COVID-19, COVID-19 symptoms, and their risk factor and found a notable occurrence of COVID-19 symptoms and their demographic relation, post-COVID-19 symptoms with statistically significant age, severity, and DM as risk factors, but still larger studies should be carried out for a longer duration to attain a deeper apprehension of the trends in symptom persistence among individuals who have experienced COVID-19 symptoms.

Study limitations

This study has some limitations. For example, the study was conducted in a very limited area which does not represent the larger community. The sample size was limited. Also, few data were purely subjective which can vary significantly among the participants of the study.

## Conclusions

This study identified that COVID-19 is most common among male adults. The most notable findings shown by this study are the significant occurrence of post-COVID-19 manifestations and establishing age, severity during acute disease, and DM as significant components for the persistence of post-COVID-19 features. Nevertheless, more research is necessary to see the association between comorbidities and clinical outcomes and the components responsible for the longer tenacious manifestations of COVID-19.

## References

[1] Diaz J, Baller A, Banerjee A (2020). Clinical Management of Severe Acute Respiratory Infection (SARI) When COVID-19 Disease Is Suspected. World Health Organization.

[2] Yoshikawa T, Hill T, Li K, Peters CJ, Tseng CT (2009). Severe acute respiratory syndrome (SARS) coronavirus-induced lung epithelial cytokines exacerbate SARS pathogenesis by modulating intrinsic functions of monocyte-derived macrophages and dendritic cells. J Virol.

[3] Fang M, Siciliano NA, Hersperger AR (2012). Perforin-dependent CD4+ T-cell cytotoxicity contributes to control a murine poxvirus infection. Proc Natl Acad Sci U S A.

[4] Zhang L, Huang B, Xia H (2020). Retrospective analysis of clinical features in 134 coronavirus disease 2019 cases. Epidemiol Infect.

[5] Yuki K, Fujiogi M, Koutsogiannaki S (2020). COVID-19 pathophysiology: a review. Clin Immunol.

[6] Yu P, Zhu J, Zhang Z, Han Y (2020). A familial cluster of infection associated with the 2019 novel coronavirus indicating potential person-to-person transmission during the incubation period. J Infect Dis.

[7] Huang R, Xia J, Chen Y, Shan C, Wu C (2020). A family cluster of SARS-CoV-2 infection involving 11 patients in Nanjing, China. Lancet Infect Dis.

[8] Pan X, Chen D, Xia Y (2020). Asymptomatic cases in a family cluster with SARS-CoV-2 infection. Lancet Infect Dis.

[9] Wei WE, Li Z, Chiew CJ, Yong SE, Toh MP, Lee VJ (2020). Presymptomatic transmission of SARS-CoV-2 - Singapore, January 23-March 16, 2020. MMWR Morb Mortal Wkly Rep.

[10] Li X, Wang L, Yan S (2020). Clinical characteristics of 25 death cases with COVID-19: a retrospective review of medical records in a single medical center, Wuhan, China. Int J Infect Dis.

[11] Hossain MA, Hossain KM, Saunders K (2021). Prevalence of long COVID symptoms in Bangladesh: a prospective inception cohort study of COVID-19 survivors. BMJ Glob Health.

[12] Fernández-de-Las-Peñas C, Palacios-Ceña D, Gómez-Mayordomo V, Cuadrado ML, Florencio LL (2021). Defining post-COVID symptoms (post-acute COVID, long COVID, persistent post-COVID): an integrative classification. Int J Environ Res Public Health.

[13] Nabavi N (2020). Long covid: how to define it and how to manage it. BMJ.

[14] (2022). Prevalence of ongoing symptoms following coronavirus (COVID-19) infection in the UK: 1 April 2021. UK: 4 June.

[15] Iwu CJ, Iwu CD, Wiysonge CS (2021). The occurrence of long COVID: a rapid review. Pan Afr Med J.

[16] Islam MS, Ferdous MZ, Islam US, Mosaddek AS, Potenza MN, Pardhan S (2021). Treatment, persistent symptoms, and depression in people infected with COVID-19 in Bangladesh. Int J Environ Res Public Health.

[17] Mahmud R, Rahman MM, Rassel MA, Monayem FB, Sayeed SK, Islam MS, Islam MM (2021). Post-COVID-19 syndrome among symptomatic COVID-19 patients: a prospective cohort study in a tertiary care center of Bangladesh. PLoS One.

[18] Gupta A, Mathur M, Padhy M, Gupta S, Solanki H, Tomar A (2021). ‘Long COVID'-frequency and pattern of persistent symptoms in COVID patients-a follow-up study from a single centre. Trends Medical Res.

[19] Rodriguez-Morales AJ, Cardona-Ospina JA, Gutiérrez-Ocampo E (2020). Clinical, laboratory and imaging features of COVID-19: a systematic review and meta-analysis. Travel Med Infect Dis.

[20] Aggarwal S, Garcia-Telles N, Aggarwal G, Lavie C, Lippi G, Henry BM (2020). Clinical features, laboratory characteristics, and outcomes of patients hospitalized with coronavirus disease 2019 (COVID-19): early report from the United States. Diagnosis (Berl).

[21] Ahmed NU, Islam MA, Kabir MA, Rahman MH, Sadat SM (2020). Clinico-pathological findings of Bangladeshi Covid 19 patients with their clinical outcome: study of a cohort of 201 cases. J Bangladesh Coll Phys Surg.

[22] Carson G (2021). Research priorities for long Covid: refined through an international multi-stakeholder forum. BMC Med.

[23] Logue JK, Franko NM, McCulloch DJ, McDonald D, Magedson A, Wolf CR, Chu HY (2021). Sequelae in adults at 6 months after COVID-19 infection. JAMA Netw Open.

[24] Huang C, Huang L, Wang Y (2021). 6-month consequences of COVID-19 in patients discharged from hospital: a cohort study. Lancet.

[25] Sudre CH, Murray B, Varsavsky T (2021). Attributes and predictors of long COVID. Nat Med.

[26] Davis HE, Assaf GS, McCorkell L (2021). Characterizing long COVID in an international cohort: 7 months of symptoms and their impact. EClinicalMedicine.

